# The Hospital Penny Fund

**Published:** 1904-10-22

**Authors:** 


### HOSPITAL ADMINISTRATION.

### CONSTRUCTION AND ECONOMICS.

### "THE HOSPITAL PENNY FUND."

### EXPLOITING THE HOSPITALS FOR PRIVATE GAIN.

#### HOW THE HOSPITALS CAN DEFEAT THE SCHEME.

We publish the further replies we have received from the hospitals, which make it clear that every reputable hospital repudiates all connection with this scheme. In view of the fact that the promoters announce their intention of exploiting 200 reputable institutions it is essential that the authorities of all our great hospitals should combine, either by conferring together and appointing representatives to publicly repudiate all connection with the scheme, or in some other way. Meanwhile, we hope that the committee and officers of every important hospital which has not already taken this step will co-operate with us in putting a stop to this scheme by at once sending us a letter intimating their position in the matter, for publication in our columns. If this be done in the course of the next fortnight it will be the most effective way of putting an end to what may otherwise become one of the gravest scandals of modern times.

#### HOW "PUBLIC OPINION" WILL BENEFIT.

*Public Opinion* of October 14, 1904, contains the following :—

Our attention has been called at the moment of going to press to quotations in the *Daily Mail* and other papers from an article to appear in this week's Hospital in which it is alleged that *Public Opinion* is to get "6⅔ per cent. of the money derived from the sale of the stamps" of the Hospital Penny Fund. The statement, whether made by The Hospital or any other paper, is an absolute fabrication, and we shall take such action as may be necessary to vindicate our connection with the Fund.

It will be observed that *Public Opinion* claims that the statement that that paper is to get 6⅔ per cent. of the money derived from the sale of the stamps is "an absolute fabrication." Is it? Let the reply be supplied by *Public Opinion.* On p. 504 of the same issue of *Public Opinion* appear two columns of particulars of what is described as "The Great Hospital Scheme. Prizes for Work." From these it appears that anyone desiring to enter for prizes must first purchase a copy of *Public Opinion.* No. 1 of the Conditions and Rules provides that "to obtain a 2s. 6d. or 1s. book of stamps you must fill in a coupon cut from *Public Opinion*, with your name and address. These coupons will be registered at the office of the Hospital Penny Fund and the prizes awarded in accordance with the published list." Under the heading "How It (the Scheme) will Work out" *Public Opinion* states : "Send to the Hospital Penny Fund for a book of 32 stamps, accompanied by 2s. 6d. and the coupon published by *Public Opinion. Public Opinion* will cost you 2d., which is the exact amount of the profit you will make on the sale of the 32 stamps. As *Public Opinion* contains two coupons send the second coupon for a second 2s. 6d. booklet when the first is exhausted, and the 2d. profit on the second transaction will recoup you for the 2d. spent on postage." *Public Opinion* adds : "If you do not care to start with 2s. 6d. start with 1s., but the 2s. 6d. is the better beginning from every point of view."

#### 6⅔ PER CENT. NOT CREDITED TO FUND.

It will be seen that every competitor has to sell to the public 32 stamps, of which only 30 are brought to the credit of the Fund. Twopence has to be paid for *Public Opinion* to enable the competitor to get a copy of that paper, and so to obtain the coupons without which he cannot compete. It is thus clear that 6⅔ per cent.—*i e*, 2d. on each 2s. 6d. booklet of stamps—though contributed by the public, cannot be credited to the Fund at all. As this 2d. comes, however, out of the pockets of the public, it must be added to the 25 per cent. retained by the promoters, making altogether 31⅔ per cent. of the total sum realised by the sale of the stamps. In practice, therefore, it does not really matter whether the 2d. goes into the pockets of the proprietors of *Public Opinion*, or to the competitor for the prizes, or to the Post Office, or in what proportions, if any, it may be divided among the three. *Public Opinion* must get a large proportion of the twopences, because no one can compete without purchasing the coupons in that paper; and, seeing the classes who are asked to compete, it is evident they are not capitalists, so the majority of them will not be likely to purchase more than one half-a-crown book at a time. In such a case *Public Opinion* will necessarily net the larger number of the twopences paid by the public, being the equivalent of 6⅔ per cent. upon the total sum realised by the sale of the stamps.

The conductors of *Public Opinion* do not appear themselves to have any fixed view as to the percentage of the total sum derived from the sale of the stamps which will ultimately find its way into the coffers of the hospitals. Thus in its issues of September 23rd, 30th, and October 7th a statement is published in large type to the effect that "out of every shilling received from the sale of these stamps ninepence goes direct to the hospitals, without any deductions whatsoever." In this week's issue (October 14th) the statement is modified to "ninepence of every shilling realised by the Fund goes to the hospitals." This is a material difference. The last statement may be correct, but the first statement is untrue, as shown by No. 1 of the Conditions, where it is stated that the 2s. 6d. book contains 32 stamps; so that the public will contribute 2d. of every 32 pence realised by the sale of these stamps which cannot be placed to the credit of the Hospitals at all.

*Public Opinion*, however, after making the correction in the first column, reproduces the misstatement in the second column of page 504 of its issue of October 14th by printing in large type these words :—"The Main Point to Remember ; 75 per cent. of all moneys received from the sale of these stamps goes direct to the hospitals." This statement is, however, untrue and misleading, as already explained. It is essential, too, that the public should know if, and to what extent, stamps are being sold to the public in addition to the two stamps attached to each 2s. 6d. book, the proceeds of which will not be brought to account in the books of the scheme.

### "TRUTH" ON "EXPLOITING THE HOSPITALS."

*Truth*, in an article in its issue of October 20, 1904, referring to this scheme declares : "The facts of this disreputable enterprise are so clear that it seems waste of time to argue about them." Dealing with the promoters' question whether *Truth* could mention any philanthropic scheme which is administered on less than, or so little as, 25 per cent. of its receipts, the Editor proceeds :—

"The question is utterly wide of the point, and could only have been put in this form for the purpose of obscuring the issue. The administration of a charity is a part of the business of the charity. In this particular case there is no charity to be administered. The administration will only begin when the money is handed over to the hospitals. What we have to look at in the Hospital Penny scheme is that 25 per cent. is sweated out of the money before it is applied to any charitable purpose, administrative or otherwise. The Penny Fund is merely a collecting agency. What I have said in relation to this particular scheme was that a charity which spent 25 per cent. of its receipts on the business of collecting money would stand self-condemned. There are dozens of dishonest burlesques of charities now in the field run on this 25 per cent. basis ; that is to say, on the basis that 5s. out of every sovereign collected goes into the pockets of the collectors. The 25 per cent. basis is the badge of the lowest form of charity-mongering."

### "A GROSS IMPERTINENCE."

*Truth* next deals with the action of *Public Opinion*.

"There is really no room for two opinions about this undertaking. If there had been, it would not have been left to an almost unknown journal of limited circulation to take it up. No one can suppose that the promoters gave the job to *Public Opinion* until they had satisfied themselves that more influential and widely-read papers were not disposed to identify themselves with it. The scheme is not inspired by charitable motives in the promoters, and does not appeal to such motives in the subscribers, except by a side wind, and the only justification that could possibly be shown for its existence is removed by what has now transpired as to the attitude of the hospitals themselves towards it. If I were to open a fund for the benefit of Mr. Henry J. Drane, proposing to retain 25 per cent. of the receipts for myself to cover my expenses in collecting the money, and if I were to take this step and set about getting in the money without obtaining Mr. Drane's sanction to my proceedings, I take it that he would regard this movement on my part as a gross impertinence, and possibly feel somewhat angry about i The movement which his journal is promoting stands precisely on the same footing."

### "AN OUTRAGE ON CHARITY."

"Had the hospitals after full consideration decided to identify themselves with this scheme, whatever one might think of the good sense of their decision, the promoters could, to a certain extent, have taken shelter behind it. But the organisation of such a collection without the fullest and most cordial approval and co-operation on the part of all the principal institutions concerned can only be accurately called, as I called it in my last article, an outrage on charity. It seems to me to be the duty of the hospitals now to take some joint action for the purpose of repudiating the scheme, and to notify their repudiation throughout the country in such a way that the public can be under no misapprehension about it. It may be, of course, that the scheme will fail ignominiously. I hope it will. But it is calculated to bring so much discredit upon the cause represented by the hospitals that these institutions, both in London and in the provinces owe it to themselves to define their attitude towards it unmistakably."

### MORE DISCLAIMERS.

### Hospital for Epilepsy and Paralysis.

The Secretary writes :—

"In reply to your letter of the 8th respecting 'the Hospital Penny Fund' I append a copy of a letter which I wrote to the Secretary of that enterprise on September 24th.

"I have only to add that I submitted the papers of the 'Fund' to my committee on the 11th reporting my letter aforesaid, and that my committee did not direct me to notice the communications any further."

H. Howgrave Graham,
October 14th, 1904. Secretary.

[Copy.]

"I will submit to my committee at their next meeting your circular of the 17th instant, and, if they so direct me, will write to you further.

"In the meantime, and until you are duly authorised to do so, you will, of course, refrain from making any use of this hospital or its name in connection with the Fund."

The Secretary, H. Howgrave Graham,
Hospital Penny Fund, Secretary.
September 24th, 1904.

### North London or University College Hospital.

The Secretary writes :—

"In reply to your letter of 8th inst., I am directed to inform you that the name of University College Hospital is being used for the purposes of the Hospital Penny Fund without the authority of the Hospital Committee."

Newton H. Nixon,
October 12th, 1904. Secretary.

### St. Thomas' Hospital.

The Secretary sends copy of the following letter to Mr. Goldman, of the Hospital Penny Fund :—

*Re* Hospital Penny Fund.

"I have put your letters, together with the printed scheme for collecting pennies to help hospitals, before the committee. The committee do not consider that it is advisable that any such Central Penny Fund should be established, and cannot therefore identify themselves with it in any way."

(Signed) G. Q. Roberts,

October 5th, 1904. Secretary.

